# Have the psychiatric needs of people seeking gender reassignment changed as their numbers increase? A register study in Finland

**DOI:** 10.1192/j.eurpsy.2023.2471

**Published:** 2023-11-06

**Authors:** Riittakerttu Kaltiala, Timo Holttinen, Katinka Tuisku

**Affiliations:** 1 Faculty of Medicine and Health Technology, Tampere University, Tampere, Finland; 2 Department of Adolescent Psychiatry, Tampere University Hospital, Tampere, Finland; 3 Vanha Vaasa Hospital, Vaasa, Finland; 4 Department of Psychiatry, Helsinki University Hospital, Helsinki, Finland

**Keywords:** epidemiology, gender dysphoria, psychiatric morbidity, register study, time-trends

## Abstract

**Background:**

The number of people seeking gender reassignment (GR) has increased everywhere and these increases particularly concern adolescents and emerging adults with female sex. It is not known whether the psychiatric needs of this population have changed alongside the demographic changes.

**Methods:**

A register-based follow-up study of individuals who contacted the nationally centralized gender identity services (GIS) in Finland in 1996–2019 (gender dysphoria [GD] group, *n* = 3665), and 8:1 age and sex-matched population controls (*n* = 29,292). The year of contacting the GIS was categorized to 5-year intervals (index periods). Psychiatric needs were assessed by specialist-level psychiatric treatment contacts in the Finnish Care Register for Hospital Care in 1994–2019.

**Results:**

The GD group had received many times more specialist-level psychiatric treatment both before and after contacting specialized GIS than had their matched controls. A marked increase over time in psychiatric needs was observed. Among the GD group, relative risk for psychiatric needs after contacting GIS increased from 3.3 among those with the first appointment in GIS during 1996–2000 to 4.6 when the first appointment in GIS was in 2016–2019. When index period and psychiatric treatment before contacting GIS were accounted for, GR patients who had and who had not proceeded to medical GR had an equal risk compared to controls of needing subsequent psychiatric treatment.

**Conclusion:**

Contacting specialized GIS is on the increase and occurs at ever younger ages and with more psychiatric needs. Manifold psychiatric needs persist regardless of medical GR.

## Introduction

Gender dysphoria (GD) in DSM-5 [1] refers to distress and impairment arising from incongruence between one’s experienced/expressed gender and primary and/or secondary sex characteristics. In ICD-10 the corresponding term is Transsexualism [2], and in ICD-11 Gender Incongruence [3]. Hormonal and surgical interventions aiming at aligning the bodily characteristics with the experienced gender are considered the treatment of choice to alleviate distress among people with sex-discordant gender identity [4]. Transgender is an umbrella term referring to all gender identities incongruent with one’s natal sex [4, 5]. Not all those identifying as transgender necessarily suffer from dysphoria or seek treatment.

The number of people seeking gender reassignment (GR) or identifying as transgender has increased throughout the Western world, these increases particularly concerning adolescents and emerging adults with female sex [6–9]. The reasons for these increases are not known; increasing awareness, reduced stigma, better service availability, and increased treatment options, but also media and social media influences and seeking belongingness have been suggested to be behind them [4, 10–13].

Transgender people in the population, in general health care settings, and specialized gender identity services (GIS) commonly present with psychological distress and mental disorders [14–18] which are often understood as secondary to stress concerning the sexed bodily characteristics or minority stress [15, 19]. However, studies notably on the youngest patients seeking GR have also reported numerous risk factors common to child and adolescent psychiatric morbidity at large, and severe mental disorders with onset preceding the onset of GD [20–23]. Among minors referred to GIS, as many as two-thirds present with diagnosable mental disorders, particularly depressive and anxiety disorders and autism spectrum conditions [17, 18]. Of adults seeking GR, about 30–40% present with current and 60–80% with lifetime diagnosable mental disorders, mainly depressive and anxiety but also substance use and personality disorders [14, 15, 24]. Most of the studies among both minors and adults have been single-center studies with small samples and without control groups. They can shed no light on possible changes in psychiatric comorbidities among people with different treatment trajectories or across times when contacts to GIS have vastly increased.

Research exploring mental health prognoses among people presenting with clinical GD and the impact of GR interventions on mental health and psychosocial functioning is rare. It is based mainly on short-term, low-quality studies, the findings being inconclusive among both adults and minors [14, 25–30]. Two studies stand out as being more informative on outcomes regarding long-term psychiatric needs: a register-based follow-up study (for an average of 10.4 years) of all 324 individuals who underwent medical GR in the period 1973–2003 in Sweden and 10:1 matched population controls [31], and a register-based study of 3754 minors with GD and their 6660 siblings from the USA [32], covering a follow-up of a mean of 8.5 years. In the former, the transgender patients had over fourfold crude risk for psychiatric morbidity during follow-up, and almost threefold when psychiatric morbidity prior to GR was accounted for [31]. In the latter study, transgender adolescents had over fivefold more psychiatric disorders than their siblings. The need for psychiatric care did not diminish after the GR interventions [32].

Thus, comprehensive, large-scale, and long-term follow-up studies on psychiatric morbidity among patients who seek medical GR are rare. Further, possible changes in these needs alongside vast increases in numbers seeking GR are so far not known. If increasing numbers of patients seeking GR relate to increased openness and treatment availability, with diminishing stigma and prejudice, mental health issues might have been expected to have likewise diminished over time in this population. A Dutch study found little change in patients admitted to child and adolescent GIS over time [33], but we are not aware of such studies among adults. Therefore, we set out to explore possible changes in the psychiatric needs of individuals seeking GR in Finland between 1996 and 2019, a period during which these contacts increased considerably. More specifically, we asked:

1. Did individuals seeking GR differ from matched population controls regarding contacts to specialist-level psychiatric treatment and disorders treated?
2. Did changes occur over time in the proportion of those having had specialist-level psychiatric treatment contacts before their first contact with GIS, and in the proportion of those needing specialist-level psychiatric care after contacting the GIS?
3. Did individuals seeking GR in Finland differ from matched population controls regarding needs for psychiatric treatment after contacting GIS, and were there differences in subsequent psychiatric needs between those who did and those who did NOT proceed to medical GR?

## Materials and methods

### Setting

Gender identity assessments potentially leading to medical GR interventions are by (code of) law [34] nationally centralized in Finland to two of the five university hospitals. Services for legal adults have been available since the early 1990s [35], and became available to minors in 2011 [20]. A doctor’s referral is required. The current national guidelines require that the necessary psychiatric assessment and treatment needs be in place before gender identity assessments can be considered [36–38], however, during the period studied there was no such threshold.

### Design

A register-based follow-up study was carried out using information routinely collected for inclusion in the nationally representative population and health care registers in Finland. Comprehensive and reliable national registers make it possible to study large patient groups and to collate information collected in different registers on an individual level using the unique personal identity code assigned to each permanent resident of Finland. Register data can be obtained for research purposes by application to the Finnish Social and Health Data Permit Authority Findata and to Statistics Finland. Data extraction, linkages, and pseudonymization are carried out by these authorities, and researchers can use the data through a special secure connection. Analyses producing overly precise information potentially enabling a person being identified must be amended to ensure the anonymity of persons included. The present study duly obtained ethical approval from the ethics committee of Tampere University Hospital (R20040R) and the relevant permissions from Findata (THL/5188/14.02.00/2020) and Statistics Finland (TK/1016/07.03.00/2020). In issuing the national guidelines for the treatment of GD for transsexual and nonbinary adults and for minors presenting with gender distress, COHERE Finland, operating under the Ministry of Social Affairs and Health, prompted comprehensive follow-up research on GR [36–38]. In accordance with Articles 6 e and 9 i and j of the Regulation (EU) 2016/679 of the European Parliament and of the Council [39], the individual informed consent of each registrar was not required.

### Data extraction

Subjects referred to GIS were identified from the hospital databases of Tampere and Helsinki University Hospitals. The first appointment in the diagnostic team in either of the two GIS was recorded as the index date. The unique personal identity numbers of the subjects thus identified, with index date and age at the index date, were securely transferred to the Finnish Social and Health Data Permit Authority Findata, where the lists were merged. In those cases when subjects had attended the gender identity units of both hospitals, the earlier index date was taken to be the index date. In total 3,665 individuals were identified as having contacted the nationally centralized gender identity units between 1996 and 2019 (=GD group).

From the Population Register, eight controls matched for age and place of residence at birth were identified for each GD group member, four males and four females. Occasionally there may not have been enough subjects to extract a group of eight controls for all cases. The final sample included 29,292 controls. The cases and the controls were followed up in registers until 9 June 2022, or until their latest specialist-level psychiatric contact was registered, whichever earlier. Mean (SD) follow-up time was 6.9(0.02) years, median 5.7 years, maximum 26.4 years.

The Population Register does not grant researchers access to information on change of registered sex in identity documents. Only registered sex at the time of data extraction is included in the data. On the extraction date, 9 June 2022, 56.1% of the GD group were legally females, and of the controls 50%.

The Care Register for Health Care (CRHC) [40] was used for information on contacts to specialist-level psychiatric services for the GD group and the controls from 1994 to 2022 (excluding contacts due to gender identity assessments). The register, in operation since 1994, records all outpatient and inpatient contacts to specialist-level health services in Finland. Dates of admission and discharge, service provider, the patient’s age at admission and diagnoses (primary and two additional) for all contacts in specialist level psychiatric services according to ICD-9 (1994–1995) and ICD-10 (1996–) were extracted and ICD-9 converted to ICD10 using WHO conversion tables [41]. The CRHC was further used to provide information on GR surgeries that were included vaginoplasty/falloplasty and mastectomy.

The register of the Social Insurance Institution of Finland (KELA) including information on prescription medications purchased and information on their reimbursement by the national social insurance [42] was used to obtain information on hormonal GR (masculinizing/feminizing hormones) in the GD group. Persons duly diagnosed with F64.0 (since 2020 also F64.8) in the nationally centralized gender identity units are entitled to a special reimbursement (code121) for their hormonal treatment when it has continued for more than a year. Patients with specified endocrine disorders are also entitled to this reimbursement.

### Measures

Sex as registered in the Population Register will be referred to below as sex or registered sex. For the controls, this invariably coincided with their biological sex.

The GD group subjects’ index date and their age at index date were assigned to all their eight personal controls.

Psychiatric treatment history other than contact to GIS was described using the following variables: any history of specialist-level psychiatric treatment (yes/no), any history of psychiatric inpatient treatment (yes/no), specialist-level psychiatric treatment (any and inpatient) before the index date (yes/no), specialist-level psychiatric treatment (any and inpatient) after the index date (yes/no).

The diagnoses recorded in specialist-level psychiatric care were used in the analyses categorized to the main diagnostic groups F00–09, F10–19, F20–29, and so forth. When necessary, in order to further anonymize the data, main categories F00–09, F10–19, and F70–79 were combined in the analyses. A diagnosis in a psychiatric main category was recorded as present if it appeared in the register as the primary or as the first or second additional diagnosis.

Medical GR interventions used in the analyses were hormonal GR (indicated by purchases of masculinizing/feminizing hormones under special reimbursement code 121) and/or mastectomy and/or vaginoplasty/falloplasty.

In order to study change over time, index dates were classified to 5-year index periods (last period 4 years) as follows: 1996–2000, 2001–2005, 2006–2010, 2011–2015, and 2016–2019.

Year of birth (continuous) was used as a covariate.

### Statistical analyses

The data were described using distributions of the categorical variables and mean(sd); median(IQR) statistics of continuous variables. Categorical variables were compared between the GD group and the controls with cross-tabulations and chi-square test/Fisher’s exact test where appropriate (Table 1). Mantel-Haenzel test was used to explore linear associations. Continuous variables were compared using *t*-test and ANOVA. Cox regression accounting for the differences in follow-up times was used to predict specialist-level psychiatric treatment after the index date. Having the latest specialist-level psychiatric contact later than the index date was used as the dependent variable. Independent variables entered were first group membership (controls vs. GD patients who had not proceeded to medical GR [GD_GR−] vs. GD patients who had proceeded to medical GR [GD_GR+]), controlling for sex and year of birth. Next, index period was added, followed by the history of specialist-level psychiatric treatment before the index date. Hazard ratios (HR) with 95% confidence intervals (95% CI) are presented. Due to the large data size, the cutoff for statistical significance was set at *p* < 0.001.

**Table 1.** Sex distribution, indicators of specialist-level psychiatric treatment and GR interventions among people seeking GR in the period 1996–2019 and their age and birth sex-matched controls

	GD group *n* = 3665	Controls *n* = 29292	*p*
Proportion females	56.1	50.0	
Any history of specialist-level psychiatric treatment	71.5	24.2	<0.001
History of psychiatric inpatient treatment	21.8	6.0	<0.001
Specialist-level psychiatric treatment before the index date	33.0	13.7	<0.001
Specialist-level psychiatric treatment after the index date	60.6	14.5	<0.001
Inpatient treatment before the index date	11.7	3.6	<0.001
Inpatient treatment after the index date	10.7	2.7	<0.001
Number (mean [SD]; median) of psychiatric treatment contacts	238.0 (9.7); 152	144.4 (6.4); 59	<0.001
Masculinizing/feminizing hormones with special reimbursement	37.1	0.1^a^	<0.001
Mastectomy	15.9	0.2^a^	<0.001
Vaginoplasty/ falloplasty	10.9	–	<0.001
Either hormonal or surgical GR interventions	38.4%	(0.1)^a^	<0.001

a Controls may have had hormonal treatments with special reimbursement for endocrine disorders and mastectomy due to medical reasons.

## Results

### Numbers and demographics

Mean (SD) age at first appointment with GIS was 24.28 (9.3) years, median(IQR) 21 (9). Across index periods, the numbers of people first seen in GIS increased, their age grew younger and the proportion of those currently female increased (Table 2). Of patients seen in GIS, 38.2% had obtained medical GR during the study period, most commonly masculinizing/feminizing hormones (Table 1).

**Table 2.** Age (mean [SD]) and current sex (%) distribution of people who contacted GIS during the different index periods

	All cases	Legal adults at index date^a^	Proportion females at the time of data extraction^b^
1996–2000 (*n* = 72)	33.9 (9.9)	34.4 (9.6)	41.7
2001–2005 (*n* = 81)	32.8 (10.9)	33.1 (10.6)	48.1
2006–2010 (*n* = 407)	27.8 (10.5)	28.1 (10.5)	48.1
2011–2015 (*n* = 1115)	24.3 (9.5)	26.0 (9.6)	45.7
2016–2019 (*n* = 1990)	22.9 (8.1)	24.9 (8.1)	64.4

a Because GIS were only officially opened to minors in 2011, development of age distribution is also presented for legal adults only across time.

b Linear by linear association, *p* < 0.001.

The GD group had more commonly needed specialist-level psychiatric treatment in general and before and after the index date than the controls and contact had on average been more intensive in the GD group (Table 1).

A linear increase across index periods was seen among both the GD group and the controls in any psychiatric treatment history and history of psychiatric treatment before the index date (Table 3). Among the GD group, a linear increase was seen in psychiatric treatment after the index date calculated over the first four index periods and the relative risk (RR) compared to that of the controls increased across periods (Table 3). Although the follow-up time was shorter in subjects referred to GIS during the later index periods, the mean number of overall psychiatric contacts and inpatient periods did not change across index periods (data not shown).

**Table 3.** Proportion (%, *n*/*N*) of patients contacting GIS during the different index periods and their age-matched controls with psychiatric treatment history and relative risk (RR) for the patients

	1996–2000	2001–2005	2006–2010	2011–2015	2016–2019	*p* (Mantel-Haenzel test for linear association)
History of specialist-level psychiatric contact						
Cases	59.7 (43/72)	61.7 (50/81)	64.6 (363/407)	71.7 (800/1115)	73.7 (1466/1990)	<0.001
Controls	16.8 (97/576)	17.8 (115/647)	22.8 (742/3254)	24.5 (2184/8911)	24.8 (3952/15904)	<0.001
*RR cases versus controls*	*3.6*	*3.5*	*2.8*	*2.9*	*3.0*	
Specialist-level psychiatric contact before the index date						
Cases	8.3 (6/72)	12.3 (10/81)	18.7 (76/407)	28.2 (314/1115)	40.3 (802/1990)	<0.001
Controls	1.2 (7/576)	5.4 (35/647)	10.6 (346/3254)	12.8 (1140/8911)	15.6 (2480/15904)	<0.001
*RR cases versus controls*	*6.9*	*2.3*	*1.8*	*2.2*	*2.6*	
Specialist-level psychiatric contact after index date						
Cases	54.2 (39/72)	56.8 (46/81)	58.0 (236/407)	63.7 (708/1115)	59.9 (1193/1990)	<0.001 /0.77^a^
Controls	16.3 (94/576)	15.3 (99/647)	16.7 (545/3254)	16.1 (1438/8911)	13.1 (2085/15904)	0.1/0.02^a^
*RR cases versus controls*	*3.3*	*3.7*	*3.5*	*4.2*	*4.6*	

a Across four first periods.

### Psychiatric diagnoses

The most common diagnosis in the first psychiatric contact was that of severe mood disorders (F30–39) in both groups, with a higher prevalence in the GD group (Table 4). The second most common diagnosis in both groups fell into the category of anxiety disorders (F40–48), with no difference between groups, and the third most common into the category of disorders with onset in childhood (F90–99). All other diagnoses were rare. The same diagnostic groups predominated in the most recent recorded psychiatric treatment contacts. (Table 4).

**Table 4.** Psychiatric diagnoses (primary or first or second additional diagnosis) recorded at the first and the last specialist-level psychiatric contact among the GD group and the controls with a history of such contact

	First psychiatric contact			Latest psychiatric contact		
	GD group *n* = 2622	Controls	*p*	GD group *n* = 2622	Controls *n* = 7090	*p*
F00–09, F10–19, F79–79	39 (1.5%)	255 (3.6%)	<0.001	67 (2.6%)	391 (5.5%)	<0.001
F20–29	50 (1.9%)	194 (2.7%)	0.01	111 (4.2%)	361 (5.1%)	0.04
F30–39	881 (33.6%)	1828 (25.8%)	<0.001	1139 (43.4%)	2368 (33.4%)	<0.001
F40–49	575 (21.9%)	1638 (23.1%)	0.1	796 (30.4%)	2303 (32.5%)	0.02
F50–59	48 (1.8%)	329 (4.6%)	<0.001	71 (2.7%)	337 (4.8%)	<0.001
F60–69	81 (3.1%)	94 (1.3%)	<0.001	241 (9.2%)	290 (4.1%)	<0.001
F80–89	91 (3.5%)	136 (1.9%)	<0.001	224 (8.5%)	281 (4.0%)	<0.001
F90–99	250 (9.5%)	791 (11.2%)	0.01	356 (13.6%	979 (13.8%)	0.4

*Note:* The first contact may have been registered with only a z-code and all subjects may have 1–3 diagnoses, thus the sums of the columns are not 100%.

Due to the small numbers presenting with any other diagnostic groups than severe mood disorders and anxiety disorders, analysis of possible changes over time in diagnostic distributions was explored dichotomizing index dates to 1996–2010 versus 2011–2019. The prevalence of the most common diagnostic groups (F30–39 and F40–48) remained stable over time in both groups. Disorders with onset in childhood (F90–99) increased in prevalence from 1996–2010 to 2011–2019 in both groups (GD group at first visit 4.8% in the earlier vs. 10.3% in the later period; controls 7.3% vs. 11.8%, respectively; GD group at the most recent visit 9.3% in the earlier vs. 14.3% in the later period, controls 7.0% vs. 14.9%, respectively; *p* < 0.001 in all comparisons). Developmental disorders (F80–89) were more commonly recorded at the most recent psychiatric contact in the later period in both the GD group (5.1% vs. 9.1%, *p* = 0.005) and the controls (1.8% vs. 4.3%, *p* < 0.001).

### Psychiatric needs and medical GR

Of those GD patients who had received GR interventions, 15.3% had psychiatric treatment contact prior to the index date, and of those who had not proceeded to medical GR, 47.0% (*p* < 0.001). Of those who had received medical GR interventions, 52.9% had had some psychiatric treatment contact subsequent to contacting GIS, and of those who had not proceeded to medical GR, 66.7% (*p* < 0.001). Among those with no psychiatric contact before the index date, subsequent need for psychiatric treatment was less common but nevertheless considerable among those receiving medical GR (49.7% vs. 56.9%, *p* < 0.001), whereas among those already needing psychiatric treatment before the index date, difference by medical GR status did not reach statistical significance (70.9% in GR+ vs. 77.8% in GR−, *p* = 0.01).

### Multivariate models

Both those GD patients who had proceeded to medical GR and those who had not were more likely to need psychiatric treatment after the index date than were the controls (Table 5). Later need for psychiatric treatment contact increased markedly in later cohorts. Psychiatric treatment before the index date was a predictor of psychiatric treatment after the index contact. When both index period and psychiatric treatment before the index date were added into the model, GD_GR− and GD_GR+ groups had an equal risk for later psychiatric treatment. In addition, psychiatric treatment after the index date was predicted by female sex and later year of birth (Table 5).

**Table 5.** Risk (Hazard Ratios [HR], 95% confidence intervals [CI]) of need for specialist-level psychiatric treatment after index date according to group membership, registered sex at data extraction, year of birth, index period, history of specialist-level psychiatric care before contacting GIS and medical GR interventions

	Model 1. Group membership, sex, and year of birth	Model 2. Group membership, sex, year of birth, and index period	Model 3. Group membership, sex, year of birth, and history of psychiatric care before the index date	Model 4. Group membership, sex, year of birth, index period, and history of psychiatric care before the index date
Group				
Controls	**Ref.**	**Ref.**	**Ref.**	**Ref.**
GD_GR−	**6.4 (6.0–6.9)**	**6.0 (5.6–6.3)**	**4.1 (3.9–4.4)**	**3.9 (3.6–4.2)**
GD_GR+	**3.6 (3.4–3.9)**	**4.0 (3.7–4.3)**	**3.5 (3.3–3.8)**	**3.8 (3.6–4.1)**
Registered sex female	**1.5 (1.4–1.5)**	**1.4 (1.4–1.5)**	**1.4 (1.3–1.5)**	**1.4 (1.3–1.4)**
Year of birth (continuous)	**1.07 (1.07–1.07)**	**1.03 (1.03–1.04)**	**1.07 (1.07–1.07)**	**1.03 (1.03–1.04)**
Index period	–		–	
1996–2000		Ref.		Ref.
2001–2005		1.6 (1.2–2.1)		1.5 (1.2–2.0)
2006–2010		**3.9 (2.3–3.9)**		**2.6 (2.0–3.4)**
2011–2015		**7.5 (5.6–9.9)**		**6.4 (4.8–8.5)**
2016–2019		**25.0 (19.7–33.5)**		**21.0 (15.7–28.2)**
Specialist-level psychiatric treatment before the index date			**3.3 (3.1–3.4)**	**3.1 (2.9–3.2)**

*Note:* ORs statistically significant at level p < 0.001 are highlighted in bold.

## Discussion

From the 1990s toward the present time, with vast increases in numbers contacting specialized GIS, the needs for psychiatric treatment among those seeking GR have increased absolutely and in relation to age and sex-matched population. The novel contributions of the present study arise from the comprehensive register data comprising a large group of unselected patients contacting specialized GIS and matched population controls as well as from the long time period covered. Earlier studies have seldom compared the psychiatric needs of GD patients with those of the general population or studied changes over time.

Over the study period, the proportion of those in contact with specialist-level psychiatric services also increased among the controls. RR in GD patients first decreased reaching its lowest before 2010, but thereafter the RR in the GD group it increased. Over time, the number of patients contacting specialized GIS increased, and they presented at younger ages. These demographic changes corroborate findings from reviews and meta-analyses on the topic [6, 8, 9]. As far as we know, the increase over time in psychiatric morbidity among those seeking GR has not previously been presented.

The most common psychiatric disorders among the GD group were severe mood disorders and anxiety disorders. This is in line with earlier research both among adults and minors [14, 17, 18]. These disorders were also most common among the controls. No statistically significant changes were seen in the proportions of these disorders over time. The increase over time in disorders with onset in childhood obviously reflects the younger index age in later cohorts. Developmental disorders (F80–89 comprising autism spectrum conditions) were more common among the GD group, which corroborates the earlier literature [43]. The increases over time in these diagnoses may reflect both actual changes in the populations studied and also increased awareness of autism.

Multivariate models taking into account differences in follow-up times between index periods showed that psychiatric needs subsequent to contacting GIS were predicted by later index period, prior psychiatric needs, and later year of birth. The need for psychiatric treatment prior to contacting GIS also explained the difference first observed in subsequent psychiatric needs between those GD patients who proceeded to medical GR and those who did not. In the final model, GD patients, regardless of GR status, continued to experience greater psychiatric treatment needs subsequent to contacting GIS than did the controls. The most readily comparable earlier study [31], likewise reported increased psychiatric morbidity after medical GR. The risk they reported was slightly less than that presented here, but then their study focused only on patients who had undergone complete medical GR while our sample included all those who contacted the GIS. The subjects in their study were older at baseline, and their sample was treated well before the contemporary increases in contacts to GIS.

Proceeding to medical GR interventions was not independent of psychiatric treatment needs prior to contacting GIS. Those who proceeded to medical GR presented less commonly with needs for specialist-level psychiatric treatment before contacting GIS and after the index date. However, of those who underwent medical GR, slightly over half had a subsequent psychiatric treatment contact, and the proportion requiring specialist-level psychiatric treatment actually increased more among those who underwent medical GR. These observations are in line with the findings reported by Hisle-Gorman et al. [32] from a register-based follow-up study where the psychiatric needs of transgender and gender-nonconforming minors were much more common than among their siblings and did not decrease after medical GD. Their findings and ours do not suggest that medical GR interventions resolve psychiatric morbidity among people experiencing gender distress.

The increase in all the younger people contacting GIS and in psychiatric needs among them have taken place simultaneously with the emergence of the widely recognized crisis in mental health among adolescents and young adults throughout the Western world [44, 45], largely associated with the increasing use of social media [44–46]. Social influences that reduce stigma and barriers to care for people suffering from incongruence between their sexed body and lived gender experience likely improve mental health in this group and social media may offer invaluable support and belongingness that buffers against minority stress. However, social media influences may also result in adolescent and emerging adult females – who present particularly frequently with identity confusion [47] – seeking for a solution to their distress through GR [11] and overshadow the need for psychiatric treatment.

### Methodological considerations

A strength of the present study is the large, nationally representative register-based sample with a sizeable group of matched population controls. Reporting to these registers is compulsory for service providers. This research focused on specialist-level psychiatric treatment contacts that reflect severe psychiatric needs. Access to specialist-level treatment requires a referral that is evaluated and accepted by the specialist-level service, and mild to moderate mental disorders are treated in primary care. Gender identity assessments that may result in proceeding to medical GR are nationally centralized to two of the five university hospitals in Finland, providing an opportunity to reliably sample the GD group seeking access to GR interventions. The long inclusion period made it possible to analyze changes over time, constituting the novel contribution of this study.

Limitations of the study include that the psychiatric diagnoses registered in CRHC may not always be based on structured diagnostic interviews. However, clinical psychiatric diagnoses among specialist-level psychiatric services have been shown to be very reliable in Finland [48]. The study does not include transgender identifying persons who have not contacted the GIS and hence cannot provide information on their psychiatric needs and changes therein. Survey studies are more suitable for exploring experiences in population not contacting health services.

This study focused on changes in psychiatric profiles among people who had contacted the GIS in order to seek medical GR. Confirming a diagnosis of Transsexualism, Gender Incongruence or GD and medical GR are possible outcomes of the assessment in the specialized GIS, but some patients will need other kinds of interventions more urgently or may nevertheless choose not to pursue medical GR. Mental health treatment may also be recommended, but specialist-level psychiatric treatment, the target of the present study, nevertheless indicates severe psychiatric disorders regardless of pathway to this level of care.

Special reimbursement for hormonal treatments due to transsexualism/GD is only granted after a year’s continuous use of hormonal treatments. Thus, patients who initiated hormonal treatments but soon discontinued will have been excluded from the group obtaining medical GR, meaning that the numbers of those initiating medical GR may have been underestimated. On the other hand, initiated but soon terminated hormone treatments likely have less impact on mental health and functioning than continued treatment.

## Conclusion

The number of people contacting specialized GIS has increased vastly since the 1990s until today, and their mean age has become steadily younger. Along with this, their needs for psychiatric treatment have increased. Both before and after contacting GIS, they present with many more common psychiatric needs than do their matched population controls, even when medical GR interventions are carried out. Among people seeking GR psychiatric needs have to be carefully assessed and addressed, also when medical GR interventions are provided. This vastly increased pursuit of GR with increases in psychiatric comorbidities warrants cautious assessment of the timeliness of medical GR and of other treatment needs that may be more urgent.
